# Motor Skill Training Promotes Sensorimotor Recovery and Increases Microtubule-Associated Protein-2 (MAP-2) Immunoreactivity in the Motor Cortex after Intracerebral Hemorrhage in the Rat

**DOI:** 10.1155/2013/159184

**Published:** 2013-07-15

**Authors:** M. V. Santos, A. S. Pagnussat, R. G. Mestriner, C. A. Netto

**Affiliations:** ^1^Post Graduate Programme in Neuroscience, Institute of Basic Health Sciences, Federal University of Rio Grande do Sul, Porto Alegre, RS, Brazil; ^2^Physiotherapy Department, Federal University of Health Sciences of Porto Alegre, UFCSPA, Rua Sarmento Leite 245, 90050-170 Porto Alegre, RS, Brazil; ^3^Faculty of Nursing, Nutrition and Physiotherapy, PUCRS, Porto Alegre, RS, Brazil; ^4^Department of Biochemistry, Federal University of Rio Grande do Sul, Porto Alegre, RS, Brazil

## Abstract

Motor skill learning may induce behavioral and neurophysiological adaptations after intracerebral hemorrhage (ICH). Learning a new motor skill is associated with dendritic reorganization and requires protein synthesis and expression of MAP-2. The purpose of this study was to evaluate motor performance and expression of MAP-2 in the motor cortex of rats submitted to intracerebral hemorrhage model (ICH) and skill task training (SK) or unskilled training (US) during 4 weeks. The Staircase test was used for behavioral evaluation, and relative optical densities and morphometrical analysis were used to estimate MAP-2 immunoreactivity and parameters of brain tissue in both motor cortices. Results show that skill task training performed with the impaired forelimb was able to increase MAP-2 immunoreactivity in the motor cortex either in sham or in ICH groups in both cortices: ipsilesional [*F*_(5,35)_ = 14.25 (*P* < 0.01)] and contralesional hemispheres [*F*_(5,35)_ = 9.70 (*P* < 0.01)]. ICH alone also increased MAP-2 immunoreactivity despite the absence of functional gains. Behavioral evaluation revealed that ICH-SK group performed better than ICH and ICH-US animals in the Staircase test. Data suggest that motor skill training induces plastic modifications in both motor cortices, either in physiological or pathological conditions and that skill motor training produces higher brain plasticity and positive functional outcomes than unskilled training after experimental intracerebral hemorrhage.

## 1. Introduction

Stroke is an important cause of persistent disability in adults [1]. Intracerebral hemorrhage (ICH) accounts from 10 to 15% of all strokes, and this cerebral vascular disease is related to a low degree of neurorehabilitation [2]. Much of the knowledge about brain plasticity mechanisms and physical rehabilitation after stroke comes from brain ischemic strokes studies. It is not possible to assume that findings in ischemic rehabilitation will apply to ICH since there are many differences in pathophysiology, location, and extension of the injury [3]. 

Upper limb motor impairment is an important functional limitation associated with diminished health-related quality of life that can persist in the long term, even with rehabilitation treatment [4]. The study of experimental stroke in animal models has provided better understanding of pathological and recovery mechanisms [5].

Experimental rehabilitative therapies may influence synaptogenesis, neurogenesis, and neuron and glial responses in addition to functional recovery [6, 7]. Although positive effects of skilled training as a rehabilitative modality have been reported, neurobiological mechanisms that support motor and functional recovery are not completely elucidated [8, 9]. Motor skill learning has the ability to induce synaptogenesis in both cortical and subcortical brain regions [10, 11], the neuroplasticity in motor cortex is related to the skill task training, and this morphophysiological changes may also mediate functional recovery after cerebral vascular disease [5]. Cellular mechanisms of plasticity and motor recovery after stroke involve coordinated neuronal changes including regulation of growth factors, increase in protein synthesis, and cortical map reorganization [5, 12]. There is evidence that the amount of microtubule-associated protein-2 (MAP-2) might be associated with sensorimotor rehabilitation in both penumbral zone and contralesional hemisphere after cerebral vascular disease [13, 14]. 

MAP-2 is one of the most important cytoskeleton proteins which is selectively located in the somatodendritic compartment of neurons and predominantly expressed in dendrites [15]. MAP-2 is considered to be a reliable marker after brain lesion. MAP-2 expression is high in the early stage after brain lesion, probably due to compensatory regeneration [16], and low in later stage after ischemia [17]. The increase of the MAP-2 expression also occurs in a neural organization induced by exercise after cerebral ischemia [18] or cerebral physiological conditions [19]. MAP-2 also regulates neuronal polarity and dendritic extension, and it promotes structure modulation and morphological stabilization in neuronal cells [15, 20]. 

The acquisition of new motor skills involves dendritic reorganization in pyramidal neurons in motor cortex which is correlated to the increase of protein synthesis and enhanced expression of MAP-2 [18]. It was demonstrated that treadmill running training improves motor function after ICH, and this improvement may be related to changes in dendritic morphology in the striatum [21]. Still, previous studies found that synapse formation in the motor cortex begins immediately when learning a new task [22]. The increase of MAP-2 expression is related to dendritic growth [23], and the increased dendritic branching is related to changes in sensorimotor behavior [24]. Although studies have shown functional changes in the upper limb and cerebral plasticity, the relationship between different motor skills practice and MAP-2 expression is not completely understood. Besides, outcomes of previous studies cannot be generalized due to the different methodological approaches.

In this study, we investigated whether different rehabilitation modalities (skilled or unskilled training) would result in a differential forelimb motor behavior and in morphological adaptations evaluated by the MAP-2 immunoreactivity in forelimb representation in motor cortex. We hypothesized that functional motor recovery and the increase of MAP-2 expression are greater after skilled as compared to unskilled training.

## 2. Materials and Methods

### 2.1. Animals

 Adult male Wistar rats (*n* = 48) were obtained from a local colony at approximately 3 months of age (300–350 g) and housed in groups of 4-5 in Plexiglas cages under standard laboratory conditions (12 h light/dark cycle with lights off 7:30 pm and controlled temperature in 22 ± 2°C). Water and standard laboratory chow were provided *ad libitum* except during behavioral adaptation, testing, and training periods. The experimental design and all procedures were approved by the Committee of Research Ethics of Federal University of Rio Grande do Sul (identifier number 2008015) and were in accordance with the Guidelines for Care and Use of Laboratory Animals adopted by the National Institute of Health (USA) and with the Federation of Brazilian Societies for Experimental Biology.

### 2.2. Behavioral Assessment

#### 2.2.1. Staircase Test

Beginning on the day before, and continuing for the duration of the Staircase test period, animals were mildly food deprived (in order to increase interest for new food). After each training session, rats were provided with a measured amount of standard laboratory chow (15–20 g) to maintain approximately 85–95% of their free-feeding weight. Three weeks before surgery (5 days per week), rats were trained to reach sucrose pellets (4.6 mm/65 mg ± 10%) in Staircase boxes (2 trials per day, 15 min each trial) [25]. This test provides a sensitive measurement of independent forelimb skilled reaching [25, 26]. Rats that did not obtain an average of 14 pellets with at least one limb over the final 2 days of training (baseline measure) were excluded from the study. Pellets from third to the seventh degree were colored differently to enhance the quantitative discrimination of the skilled reaching [27]. Testing sessions consisted in 4 trials (two trials per day with at least 4 hours interval, 15 min each) conduced before (baseline) and after surgery (postsurgery, week 2 and week 4). 

#### 2.2.2. Intracerebral Hemorrhage Surgery

Animals were anesthetized with 4% halothane in 30% oxygen and 70% nitrous oxide and maintained in a stereotaxic frame with 2% halothane for ICH surgery. A midline incision was made in the scalp, and a burr hole was drilled in the skull 3.6 mm lateral to Bregma. Surgery side was contralateral to the preferred paw, which was determined according to Staircase evaluation (baseline). Then, a 26-gauge needle (Hamilton, Reno, NV, USA) was inserted 6.0 mm deep into the hole, and 0.2 U of bacterial collagenase type IV (Sigma-Aldrich, USA) diluted in 1.0 *μ*L saline buffer was infused into the striatum [28] over 5 min. The needle was kept in position for an additional 5 min and then slowly removed to prevent backflow. In sham surgeries, collagenase was replaced with sterile saline. Body temperature was maintained between 36.5°C and 37.5°C throughout the surgery using a self-regulating heating blanket (Letica, Spain). A local anesthetic (Lidocaine, 3M, Brazil) was applied to the wound at the end of surgery. Animals were allowed to rest during 4 days after sham or ICH surgeries. 

#### 2.2.3. Skilled and Unskilled Training

Animals received one session for habituation in skilled and unskilled tasks prior to surgery. Before the surgery, rats were grouped according to their reaching success (baseline in the Staircase evaluation) and randomly designated to one of six groups: sham no task (S, *N* = 08), sham skilled task (S-SK, *N* = 08), sham unskilled task (S-US, *N* = 08), ICH no task (ICH, *N* = 08), ICH skilled task (ICH-SK, *N* = 08), and ICH unskilled task (ICH-US, *N* = 08). Seven days after sham/ICH surgery, S-SK and ICH-SK rats received daily skilled reaching session (5 days per week). These animals were removed from their housing cages and placed into standard rodent cages containing a Plexiglass reaching apparatus [13]. The shelf below to the unaffected forelimb was left empty, whereas the shelf below to the impaired forelimb was filled with the same sucrose pellets used for Staircase test (in a sufficient amount to prevent tongue and unaffected forelimb retrieval). Animals had access to sugar pellets (15 g) for 40 min per day, and the total of pellets retrieved was measured at the end of each session (measurement by total weight). During the session, rats had free access to water, but no other type of food was available. At the end of each training day, the S, S-US, ICH, and ICH-US animals received the same average of pellets provided for S-SK and ICH-SK rats. For unskilled training, S-US and ICH-US groups received daily walking session (5 days per week) on adapted motorized rodent treadmill (INBRAMED TK 01, Porto Alegre, Brazil). Each session consisted of walking at speed 1.8 m/min during all time (40 min per day) [8]. This session duration was chosen to maximize the comparison among all groups, and the slow speed was selected to incentive animals to walk (not to run) and to limit the possible effects of aerobic conditioning. The grade of the treadmill remained at 0%, and no aversive stimulus was used.

### 2.3. Histological Analyses

After 4 weeks, animals were deeply anesthetized with chloride hydrate (30%, 10 mL/kg, i.p.) and injected with 1000 UI heparin (Cristália, Brazil). Then, animals were perfused through the left ventricle, using a peristaltic pump (Control Company, São Paulo, Brazil) with 200 mL of saline solution 0.9% followed by 150 mL of fixative solution composed of 4% paraformaldehyde (PFA) (Reagen, Rio de Janeiro, Brazil) in 0.1 M phosphate buffer (PBS) pH 7.4 at room temperature. Brains were postfixed in 4% PFA at room temperature for 4 h, kept in 30% sucrose in PBS 0.1 M at −4°C for 3 days, and then frozen in isopentane and liquid nitrogen. Coronal sections (40 *μ*m) were obtained using a cryostat (Leica, Germany). Slices obtained from the motor cortex were selected according to Paxinos and Watson [29] and stored in a solution of freezing (40% PBS 0.1 M pH 7.4; 30% ethylene glycol; and 30% glycol) until the immunohistochemistry staining procedure.

Immunohistochemistry staining was performed for MAP-2 (M9942, Sigma, USA, monoclonal produced in mouse). Briefly, free-floating sections were washed in PBS, fixed in 4% PFA, pretreated with cooled 100% methanol in 3% H_2_O_2_, and then carefully washed and blocked with 5% normal goat serum (NGS) (G9023, Sigma, USA) in PBS containing 0.3% Triton X-100 (PBSTx, T9284, Sigma Aldrich, USA) for 30 min. Brain slices were then incubated with primary antibody diluted in PBS-Tx (1 : 1000) overnight at 4°C. Sections were washed in PBS and incubated in goat anti-mouse biotinylated secondary antibody (1 : 500) diluted in 0.3% PBS-TX and 5% NGS for 1 hour at room temperature. After slices had been washed in PBS, they were treated along 30 min with Kit Vectastain ABC Elite (Vector Labs), washed in PBS, and exposed for 5 min in 3.3 diaminobenzidine (DAB, D8001, Sigma, USA) and H_2_O_2_. Sections were raised, mounted on gelatinized slides, dehydrated with ethanol, cleared in xylene, and covered with DPX mount (Sigma) and coverslips. Control sections were simultaneously processed without the primary antibody addition in order to serve as a background control [30]. Brain slices were analyzed (400x magnification) in a Nikon Eclipse E-600 microscope (Japan) coupled to a Nikon DXM 1200C CCD camera and to NIS Elements AR 2.30 software. 

The estimation of hemispheric and striatal areas as well as the cortical thickness (ipsilesional and contralesional to injury) was conducted in one slice per animal (*n* = 5 for each experimental group) in the same brain slices used in the immunohistochemistry staining procedure. These measurements were performed at the level +2.04/Interaural 11.04 mm from bregma [29]. Images were scanned in 1200 dpi, and Scion Image J 4.0 program (Scion Corporation, Frederick, MD, USA) [31, 32] was used for the morphometric analysis. Estimation of cortical thickness was calculated using the average of three measurements obtained from the upper edge of the corpus callosum until the upper edge of the hemispheres. 

For MAP analyses, two motor cortex images were used between M1 and M2 (at the level +2.16 to −0.36 mm from bregma) preferably in layer V, and cortical thicknesses were captured from both hemispheres (6 animals per group). The intensity of MAP-2 immunoreactivity was measured by relative optical densities. Digitized images were converted to an 8-bit gray scale (256 gray levels). Picture elements (pixels) employed to measure relative optical densities were obtained from region of interest (ROI) squares with 82.145,43 *μ*m² overlaid on the gray scale image, with background correction. All lighting conditions and magnifications were kept constant during the capture process.

### 2.4. Statistical Analysis

Data normality distribution was tested with the Kolmogorov-Smirnov test. Behavioral data were analyzed by one-way repeated measures analysis of variance (ANOVA). Morphometric measurements and MAP-2 relative optical densities were analyzed by one-way ANOVA followed by Tukey post hoc tests when appropriated. Data were reported as mean ± standard error of the mean (SEM). Results were considered significant when *P* ≤ 0.05. SPSS 16.0 (Statistical Package for the Social Sciences, Inc., Chicago, USA) was used for data analysis.

## 3. Results

All ICH rats showed motor behavior compatible with surgery success, which included spontaneous rotation towards to the contralateral surgery side when held by the tail [28]. A total number of animals were used for behavioral analysis (*n* = 48), and 36 animals were randomly chosen for morphological assessment (6 animals per group).

### 3.1. Staircase Test

The Staircase test analysis revealed a significant effect of “time” [*F*_(3,126)_ = 114.77, *P* < 0.01], effect of “group” [*F*_(1,42)_ = 8.22, *P* < 0.01], and “time × group” interaction [*F*_(15,126)_ = 20.78, *P* < 0.01]. Baseline evaluation performed before surgery showed no difference between experimental groups (*P* > 0.05). All ICH groups retrieved significantly fewer pellets than S animals in postsurgery and 2nd and 4th week evaluations (*P* < 0.01). There was no difference between the number of pellets collected by S-SK and S-UK animals at any point (*P* > 0.05). Tukey post hoc tests showed difference between ICH and ICH-SK and between ICH-SK and ICH-US groups (*P* < 0.01) in the 4th week. At this point, there was no difference between ICH and ICH-US groups (*P* > 0.05) as shown in Table 1.

**Figure 1:**
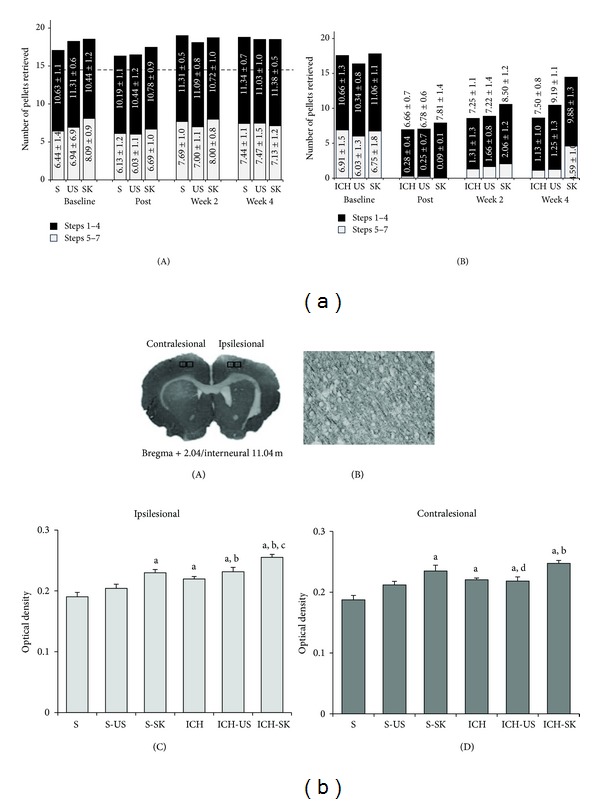
(a) Graphic representation of numbers of pellets consumed. Pellets from the first steps (1st to 4th) and from deeper steps (5th to 7th) over time. S (sham), ICH (intracerebral hemorrhage), SK (skilled training), and US (unskilled training). Data are presented as mean ± standard error (SEM). Dotted line represents minimum criteria of pellets retrieved for inclusion of animals in the study. Data are presented as mean ± standard error (SEM). (b) (A) Representative brain slice stained with MAP-2 showing areas of the interest (AOI) used to determine M1 optical density. (B) Representative area showing MAP-2 staining. (C) Relative optical density of MAP-2 staining in ipsilesional M1: “a” differences between S and S-SK (*P* < 0.001), ICH (*P* < 0.02), ICH-SK, and ICH-US (*P* < 0.001); “b” difference between S-US and ICH-SK (*P* < 0.001), and ICH-US (*P* < 0.03); “c” difference between ICH and ICH-SK (*P* < 0.01). (D) Relative optical density of MAP-2 staining in contralesional M1: “a” difference between S and S-SK, ICH, ICH-SK (*P* < 0.01), and ICH-US (*P* < 0.02); “b” difference between S-US and ICH-SK (*P* < 0.01); “d” difference between ICH-SK and ICH-US (*P* = 0.04). S (sham), ICH (intracerebral hemorrhage), SK (skilled training), and US (unskilled training). Data are presented as mean ± standard error (SEM).

For pellets retrieved from deeper stairs (5th to 7th), repeated measures ANOVA showed “time” [*F*_(3,126)_ = 94.27, *P* < 0.01], “group” [*F*_(5,42)_ = 77.73, *P* < 0.01], and “time × group” interaction effects [*F*_(15,126)_ = 17.38, *P* < 0.01]. Differences were evidenced among ICH and all sham groups (*P* < 0.01) in all evaluation times. The last point measurement (4th week) revealed difference between ICH and ICH-SK and between ICH-SK and ICH-US groups (*P* < 0.01) as depicted in Figure 1(a) and Table 2.

### 3.2. Morphometric Analysis

Collagenase injection resulted in extensive damage to dorsolateral striatum area and adjacent tissue. One-way ANOVA of morphometric data showed significant main effects on total hemisphere area [*F*_(5,29)_ = 24.27, *P* < 0.01], lesional area [*F*_(5,29)_ = 19.35, *P* < 0.01], and cortical thickness [*F*_(5,29)_ = 35.18, *P* < 0.01] only in the injured hemisphere. Differences among all sham and ICH groups were revealed by Tukey post hoc tests (*P* < 0.01). As displayed in Table 3, no task-related effects on morphometric data were found (*P* > 0.05).

### 3.3. MAP-2 Relative Optical Density Analysis

Quantitative results from MAP-2 relative optical densities are depicted in Figure 1(b). There was a significant effect of “group” in both hemispheres, ipsilesional (injured side) [*F*_(5,35)_ = 14.25 (*P* < 0.01)], and contralesional side [*F*_(5,35)_ = 9.70 (*P* < 0.01)]. 

Differences among S and S-SK (*P* < 0.001), ICH (*P* = 0.02), S-US, ICH-US, and ICH-SK (*P* < 0.001) and between S-US and ICH-US (*P* = 0.03), ICH, and ICH-SK (*P* < 0.01) groups were found in the ipsilesional hemisphere. 

When the contralesional hemisphere was analyzed, differences were found among S and S-SK, ICH, ICH-SK (*P* < 0.01), and ICH-US (*P* = 0.02) and between S-US and ICH-SK (*P* < 0.01), ICH-SK, and ICH-US (*P* = 0.04). 

## 4. Discussion

The purpose of the current study was to investigate whether different rehabilitation modalities (skilled or unskilled training) would result in a differential upper limb motor behavior and in morphological adaptations as evaluated by the means of MAP-2 immunoreactivity in the forelimb representation in motor cortex. The significance of investigating this approach refers to the major limitations of recovery after stroke and functional disability of upper limb [4, 33]. The persistence of this deficit impacts directly in an index of independence and quality of life [34]. Present results show that skill task training performed with the impaired forelimb was able to increase MAP-2 immunoreactivity in motor cortex either in sham or in ICH groups in both cortices. ICH alone also increased MAP-2 immunoreactivity despite the absence of functional gains. Behavioral evaluation revealed that ICH-SK performed better than ICH and ICH-UN animals in the Staircase test.

Physical motion is between therapeutic interventions that can improve brain recovery after stroke [3, 12]. The type of treatment, intensity and duration of the protocol, and the period in which it is applied are factors related to beneficial effects on functional recovery [35–37]. Staircase test was used to provide the quantification of the motor ability [26]. This analysis revealed that after ICH, only animals submitted to skill task training showed significant improvement in motor performance at the end of the 4th week of treatment, as evidenced by the total number of retrieved pellets. Color-coded pellets can be used in Staircase test aiming to increase the sensitivity of the instrument since it allows taking into consideration the levels of reaching difficulty [27]. All ICH animals exhibit difficulties in reaching pellets from the last steps (5th to 7th) immediately after ischemia and at the end of the 2nd week of treatment. ICH-SK group presented superior reaching rate when compared to ICH and ICH-US groups at the end of the 4th week. This may indicate that skill task training is able to induce superior upper limb recovery after intracerebral hemorrhage when compared to unskilled task training. In agreement with our results, another study demonstrated that skilled reaching training of impaired forelimb can induce brain plasticity which was observed in association with enhanced sensorimotor recovery after stroke in sensorimotor cortex and striatum [13, 38, 39]. 

Previous investigations also have found that activity-based therapies to promote forelimb use after stroke are able to develop cortical adaptations as protein synthesis, synaptogenesis, and increase in cortical representation of hand and fingers [12, 37]. These modifications correlated with functional recovery are usually proportional to the complexity of the task and do not occur in the same intensity when movement or gestures are executed in a simple and repetitive pattern [10, 36, 40]. Our behavioral results showed that physical motion can induce motor recovery, but that skill task training as a rehabilitative strategy has the capacity to improve motor performance [4]. Attention and motivation are considered essential elements in a rehabilitation program [9]. Therefore, food obtained as a reward may be considered a motivational factor that contributes for morphophysiological changes associated with behavioral responses [13, 14].

Even though behavioral assessment evidenced motor recovery, morphometric analysis showed differences only between injured and uninjured animals. Histological and biochemical studies have demonstrated a mild correlation between motor performance and these measurements in collagenase-induced intrastriatal hemorrhage and focal ischemic models [8, 29]. Our finding reinforces the idea that morphometrical parameters cannot be applied as a prognostic factor of motor recovery in experimental studies. In view of the lack of correlation between morphometrical/macroscopic analysis and behavioral results, probably the supporting for behavioral changes observed in our results is due to protein synthesis and synaptogenesis [17].

MAP-2 immunoreactivity in the motor cortex was increased by skilled task training in both cerebral hemispheres either in physiological or pathological (ICH) conditions. Motor learning is a set of internal processes associated with practice or experience that involves a coordinate system in which a planned task is encoded and combined with appropriate recruitment of motor units to execute a movement goal [41, 42]. The synaptogenesis induced by motor practice or synaptic reinforcing transmission may consolidate the motor gesture learned [43] in both intact and stroke-injured brains [11, 36]. However, our behavioral and morphological results suggest that the ICH model is adequate for future studies regarding brain injury and motor recovery by greater understanding between the morphological changes of the nervous cells and motor neurorehabilitation techniques.

MAP-2 is a structural protein that acts with other intracellular components to maintain neuroarchitecture, preferably in dendrites [44]. Furthermore, MAP-2 plays in the growth, differentiation, and plasticity of neurons, with key roles in neuronal responses to growth factors, neurotransmitters, synaptic activity, and neurotoxins [30, 45]. The increase of MAP-2-positive dendrites is connected with neural activity regeneration after injury cerebrovascular [17, 46], and dendritic morphological changes may be involved in motor learning [21]. Since modification and rearrangement of MAP-2 are obligatory steps in many processes that modify neuronal function, we decided to investigate the effects of skilled and unskilled training after ICH on functional recovery and MAP-2 expression.

The motor recovery after ischemia is influenced by the regeneration of neuritis [47]. Techniques for inducing restoration of excitability in motor cortex can exert positive effects on rehabilitation therapy by means of changes in dendritic structure and expression of MAP-2 [23, 48]. Dendritic growth process seems to be related to the amount of motor cortex inputs [49] and partially depends on MAP-2 expression [14]. 

Relative optical densities analysis revealed that the amount of MAP-2 was significantly higher in ICH animals submitted to skill task training (ICH-SK) when compared to ICH and ICH-UN in the ipsilesional and contralesional cortices, respectively. Even though ICH and ICH-US groups have shown increased MAP-2 immunoreactivity in both ipsi- and contralesional motor cortices, no behavioral improving outcome was observed. Since MAP-2 is involved with neuronal plasticity under physiological and pathological conditions, those levels of immunoreactivity after ICH were not surprising. On the other hand, motor cortex lesion causes damage in cortical networks and their projections for movement and results in contralateral limb disuse. Compensatory use of the unaffected limb can be followed by the increasing of the cortical activity in the contralesional hemisphere [48], which can explain levels of MAP-2 immunoreactivity in contralesional cortex in ICH nonrehabilitation group. 

Rehabilitation promotes motor recovery after stroke, but many patients never regain total independence. This limitation can be related to the strategy chosen as rehabilitative program since each approach can stimulate different patterns of CNS plasticity [39, 50]. Our results reinforce previous findings and reaffirm that skill task training is a good option for rehabilitation after stroke as demonstrated by the behavioral motor recovery and morphological changes.

## 5. Conclusion

Our main results demonstrated that skill task can drive brain plasticity in motor cortex by the increasing of MAP-2 expression in both hemispheres and with positive functional outcomes after collagenase-induced intrastriatal unilateral hemorrhage in a superior manner when compared to unskilled training. These findings may offer insights for improving functional recovery in stroke patients since that rehabilitation depends on type, intensity, and duration of approaches used as physical treatment. 

## Figures and Tables

**Table 1:** Number of total reached pellets in the Staircase test over time.

Groups	Baseline		Post		Week 2		Week 4	
	Mean	SEM	Mean	SEM	Mean	SEM	Mean	SEM
S	17.06	0.82	16.31	0.65	19.00	0.51	18.78	0.54
S-US	18.25	0.74	16.47	0.44	18.09	0.65	18.50	0.46
S-SK	18.53	0.53	17.47	0.24	18.72	0.56	18.50	0.57
ICH	17.56	0.90	6.94^a^	0.24	8.56^a^	0.87	8.63^a^	0.61
ICH-US	16.38	0.61	7.03^a^	0.31	8.88^a^	0.39	10.44^a^	0.27
ICH-SK	17.81	0.90	7.90^a^	0.56	10.56^a^	0.78	14.47^a,b,c^	0.63

^a^Difference between uninjured and injured groups (*P* < 0.01); ^b^difference between ICH-SK and ICH groups (*P* < 0.001); and ^c^difference between ICH-SK and ICH-US groups (*P* < 0.001). S (sham), ICH (intracerebral hemorrhage), SK (skilled training), and US (unskilled training). Data are presented as mean ± standard error (SEM).

**Table 2:** Number of reached pellets from deeper steps (5th to 7th) in the Staircase test over the time.

Groups	Baseline		Post		Week 2		Week 4	
	Mean	SEM	Mean	SEM	Mean	SEM	Mean	SEM
S	6.44	0.52	6.13	0.43	7.69	0.37	7.44	0.42
S-US	6.94	0.63	6.03	0.42	7.00	0.40	7.47	0.55
S-SK	8.09	0.32	6.69	0.38	8.00	0.30	7.13	0.44
ICH	6.91	0.55	0.28^a^	0.15	1.31^a^	0.49	1.13^a^	0.36
ICH-US	6.03	0.48	0.25^a^	0.25	1.66^a^	0.30	1.25^a^	0.47
ICH-SK	6.75	0.64	0.09^a^	0.07	2.06^a^	0.45	4.59^a,b,c^	0.36

^a^Difference between uninjured and injured groups (*P* < 0.001); ^b^difference between ICH-SK and ICH groups (*P* < 0.001); and ^c^difference between ICH-SK and ICH-US groups (*P* < 0.001). S (sham), ICH (intracerebral hemorrhage), SK (skilled training), and US (unskilled training). Data are presented as mean ± standard error (SEM).

**Table 3:** Data obtained from morphometric analysis in ipsilesional and contralesional hemispheres, cortical thickness, and cortical area.

Groups	Hemispheric area		Cortical thickness		Lesion area
	Ipsilateral	Contralateral	Ipsilateral	Contralateral	Ipsilateral
S	38.05 ± 1.10	37.08 ± 0.80	1.65 ± 0.01	1.64 ± 0.03	0
S-US	36.46 ± 1.171	37.1 ± 1.19	1.66 ± 0.01	1.63 ± 0.01	0
S-SK	40.59 ± 0.96	39.63 ± 1.75	1.74 ± 0.04	1.70 ± 0.04	0
ICH	28.29 ± 0.48*	30.28 ± 0.65	1.31 ± 0.05*	1.64 ± 0.03	4.18 ± 0.11*
ICH-US	29.09 ± 1.43*	33.1 ± 0.54	1.14 ± 0.04*	1.57 ± 0.02	4.67 ± 1.26*
ICH-SK	29.28 ± 1.15*	33.77 ± 0.51	1.28 ± 0.05*	1.45 ± 0.02	3.76 ± 0.22*

*Difference between uninjured and injured groups (*P* < 0.01). S (sham), ICH (intracerebral hemorrhage), SK (skilled training), and US (unskilled training). Data are presented as mean ± standard error (SEM).

## Glossary

ICH: Intracerebral hemorrhage
SK: Skilled task training
US: Unskilled training
MAP-2: Microtubule-associated protein-2
S: Sham: no task training
S-SK: Sham skilled task
S-US: Sham unskilled task
ICH: Intracerebral hemorrhage: no task training
ICH-SK: ICH skilled task
ICH-US: ICH unskilled task
CNS: Central nervous system.
